# Palmitic acid promotes human retinal pigment epithelial cells migration by upregulating miR-222 expression and inhibiting NUMB

**DOI:** 10.18632/aging.204647

**Published:** 2023-04-13

**Authors:** Fengzhi Li, Chunling Lei, Ke Gong, Shuwei Bai, Lianyi Sun

**Affiliations:** 1 Shaanxi Eye Hospital, Xi’an People’s Hospital (Xi’an Fourth Hospital), Affiliated Guangren Hospital, School of Medicine, Xi’an Jiaotong University, Xi’an, Shaanxi 710004, China

**Keywords:** palmitic acid, retinal pigment epithelial cells, migration, epithelial-mesenchymal transition, miR-222, NUMB

## Abstract

High glucose promotes retinal pigment epithelial cell (RPEC) migration. However, the underlying molecular mechanisms explaining how high fatty acid levels affect RPEC migration remain largely unknown. We investigated whether and how palmitic acid (PA) impacts the migration of human RPEC cell line ARPE-19. ARPE-19 cells were treated with varying doses of palmitic acid, and the RPEC migration was evaluated by scratch and transwell migration assays. Cell viability was determined by the CCK-8 method. The levels of epithelial-mesenchymal transition (EMT)-associated proteins, including E-cadherin, vimentin, MMP2, and MMP3, were evaluated by western blot. The microRNAs and mRNAs levels were assessed by quantitative PCR. miRNA targets were predicted with online tools and validated with the luciferase reporter assay. miRNA mimics, inhibitors, and siRNA oligos were used to perform gain-of-function and loss-of-function studies. We found that PA increased viability of ARPE-19 cells, promoted their migration and EMT. PA decreased E-cadherin protein expression, and increased vimentin, MMP2, and MMP3 protein levels. Additionally, PA increased miR-222 expression in ARPE-19 cells, and functionally blocking miR-222 suppressed the PA-induced RPEC migration and EMT. NUMB was identified as a downstream target of miR-222, and NUMB knockdown abolished the effects of PA on promoting the migration and EMT of ARPE-19 cells. Therefore, PA promotes human RPEC migration by upregulating miR-222 expression and downregulating NUMB. This study unravels a novel PA-miR-222-NUMB axis that can be potentially targeted for therapy of high fat acid-related ocular diseases.

## INTRODUCTION

Retinal pigment epithelial cells (RPECs) create a formidable blood-retina barrier located between the neural retina and choroid, which are essential for supporting normal and efficient photoreceptor functions [1, 2]. RPECs are usually mitotically inactive, and newly generated peripheral RPECs are required to replace dead RPECs at the central retinal pigment epithelium (RPE) due to damage and aging [3, 4]. Therefore, RPEC migration and proliferation are important steps for maintaining RPE functional integrity under physiological conditions and various ocular disease conditions, including proliferative vitreoretinopathy (PVR) and age-related macular degeneration (AMD) [5, 6]. In PVR, activated RPECs migrate into the vitreous and on the retinal surface, and their subsequent proliferation and membrane formation result in emerging fibrous membranes, whose contracting force can eventually lead to retinal detachment [7]. In AMD, RPEC intraretinal migration into the neurosensory retina has been reported as an important chorioretinal atrophy and RPEC death precursor [8–10]. In addition, RPEC intraretinal migrations occur in various AMD stages, and more commonly in eyes with serous and drusenoid pigment epithelial detachment [10]. Thus, a fine-tuned control of RPEC migration is critical for RPEC function, under both normal and disease conditions. However, the molecular pathways responsible for RPEC migration are not clearly understood.

During some pathological processes, RPECs often lose epithelial characteristics through an epithelial-mesenchymal transition (EMT), and transform into mesenchymal phenotypes and causing increased RPEC migration [11, 12]. EMT is characterized by decreased epithelial protein expression, such as E-cadherin, and increased mesenchymal protein expression, such as vimentin [13]. Additionally, upregulated matrix metalloproteinase-2 (MMP2) and MMP3 expression, enzymes that facilitate cell migration and invasion, play an important role in the EMT process in multiple cell types, including RPECs [14–16]. RPE functions mainly in protecting the photoreceptors from photo-oxidation and phagocytosis, as well as transporting water and nutrients, such as glucose, retinol, and fatty acids [17]. High glucose activates and dysregulates various metabolic pathways, and promotes RPEC migration through increased oxidative stress and pigment epithelium-derived factor (PEDF) expression in a diabetic retinopathy animal model [18]. In addition to hyperglycemia, type 2 diabetes mellitus (T2DM) associated insulin resistance is also well correlated with excessive amounts of free fatty acids (FFAs) [19]. Palmitic acid (PA), the most common saturated FFA found in the human body, represents 20%–30% of the total fatty acids in membrane phospholipids [20]. However, how PA affects human RPEC migration is not elucidated so far.

MicroRNAs (miRNAs) are a group of small non-coding RNA molecules that consist of approximately 23 nucleotide pairs. They exert profound effects on gene expression regulation by modulating target mRNA stability and/or translation efficiency [21]. miRNA roles in RPEC survival, differentiation, and function have been previously described [22, 23]. However, how miRNAs contribute to RPEC migration is still unclear. For example, miR-125b and let-7a were recently identified to promote RPEC maturation by analyzing global miRNA expression patterns during human embryonic stem cell differentiation into RPE [24]. In addition, miR-27a downregulates Toll-like receptor 4 (TLR4) expression in human RPECs when exposed to high glucose, suggesting a miR-27a protective role in inhibiting RPEC inflammation and apoptosis during hyperglycemia-mediated diabetic retinopathy pathogenesis [25]. Nevertheless, the miRNAs and underlying molecular mechanisms regulating the RPEC response to high fatty acid levels, like PA, remain obscure until now. In addition, how miR-222, a microRNA that promotes cell migration and EMT in multiple tumor types [26–29], functions in RPEC migration is not known.

As an endocytic adaptor protein, NUMB (NUMB endocytic adaptor protein) localizes to the basement layer of polarized epithelial cells and mediates endocytosis and cell membrane protein transport [30]. In rat retina, asymmetric segregation of Numb, resulting in unbalanced Notch signaling in the two daughter cells, was found to be critical for RPE development [31]. In addition, the antagonism between Numb/Numbl and Opo in zebrafish and immortalized human pigment epithelial cells RPE-1 controlled retinal epithelium morphogenesis by regulating integrin endocytosis [32]. However, whether NUMB, which plays roles in cell adhesion, migration, and polarity of RPEC [30–32], is involved in palmitic acid-regulated RPEC migration remains to be elucidated.

In this study, we evaluated the impacts of PA on RPEC migration using a spontaneously immortalized human RPEC cell line ARPE-19, and identified miR-222 responsible for palmitic acid-mediated changes in RPEC migration and EMT. In addition, a small-scale screening was performed to identify the downstream target mRNA in mediating palmitic acid’s effect on human RPECs.

## MATERIALS AND METHODS

### Cell culture and treatments

The Adult Retinal Pigment Epithelium-19 (ARPE-19) cell line was obtained from the American Type Cell Collection (ATCC^®^ CRL-2302™, Manassas, VA, USA). This cell line was authenticated by Short Tandem Repeat DNA profiling and tested as mycoplasma contamination-free by the vendor. Cells were maintained in complete Dulbecco’s Modified Eagle Medium (DMEM/F12) supplemented with 200 mM L-glutamine, 15 mM HEPES, 10% fetal bovine serum (HyClone™, Thermo Fisher Scientific, Waltham, MA, USA), 100 units/ml of penicillin and 100 μg/ml of streptomycin (Gibco; Thermo Fisher Scientific, Waltham, MA, USA). Cells were grown in a humidified atmosphere with 5% CO_2_ at 37°C. The culture medium was renewed 2 to 3 times weekly, and cells were sub-cultivated after trypsinization. PA was purchased from Sigma-Aldrich (St. Louis, MO, USA), and dissolved in ethanol at a stock concentration of 400 mM. ARPE-19 cells were treated with PA at the indicated concentrations for 48 h.

### miRNA mimic, inhibitor, and siRNA transfection

The miRNA mimics (miR-222-5p and others), inhibitors, small interfering RNA (siRNA) oligos against NUMB, and their corresponding negative controls (NC) were chemically synthesized by Shanghai GenePharma Co., Ltd. (Shanghai, China). The nucleotide sequences are listed in Supplementary Table 1. ARPE-19 cells in the logarithmic growth phase were inoculated in 24-well plates at a density of 5 × 10^4^/ml and cultured for 24 h. Cells were transfected with the indicated miRNA mimics, inhibitors, or siRNA oligos with Lipofectamine 3000 Transfection Reagent (Thermo Fisher Scientific, Waltham, MA, USA) following the manufacturer’s instructions. Cells were subjected to further analyses 48 h after transfection.

### Cell viability assay

Cell viability was analyzed by using the Cell Counting Assay Kit-8 (CCK-8; Dojindo Molecular Technologies, Inc., Kumamoto, Japan) according to the manufacturer’s protocol. Briefly, treated cells were cultured in 96-well flat-bottomed microplates, and incubated in 10% CCK-8 diluted in normal culture medium for 1 h at 37°C. The absorbance of each well at 490 nm was measured using a microplate reader (Bio-Rad Laboratories, Hercules, CA, USA). Each experiment was repeated three times with technical triplicates for each group.

### Scratch assay

ARPE-19 cells seeded into 6-well plates were left un-transfected or transfected with the indicated miRNA mimics, inhibitors, or siRNA oligos. Cells at ~100% confluency were scratched using a 1 ml micropipette tip 48 h after transfection. The detached cells were removed after washing twice with growth medium. Cells were further cultured in the medium with or without PA for 48 h. Wound closure was monitored by phase microscopy at different time points (0 h and 48 h), and images were captured in digital format. The migrated distance, as a percentage of total distance, was determined for relative wound closure.

### Transwell migration assay

ARPE-19 cells were transfected with miRNA mimics, inhibitors, or siRNA oligos for 48 hours. After, the cells were trypsinized and resuspended in serum-free DMEM medium containing PA at the indicated concentrations. Cells (1 × 10^5^) in 0.1 ml of serum-free medium were added to top pre-treated Corning^®^ Costar transwell inserts (8 μm pore size, 6.5 mm membrane). The inserts were placed in a 24-well plate containing 0.5 ml of serum-free medium and incubated for 4 h at 37°C. After incubation, the migrated cells on the lower surface of the membrane were fixed in 1% paraformaldehyde and stained with crystal violet (Sigma-Aldrich, St. Louis, USA). Cells were visualized and counted under an inverted microscope. Cells were imaged using low power (100×) magnification, and five visual fields per group were randomly selected for cell counting.

### Reverse transcription-quantitative PCR (RT-qPCR)

Total RNA was isolated from ARPE-19 cells using TRIzol (Thermo Fisher Scientific) according to the manufacturer’s instructions. Reverse transcription was performed using PrimeScript^®^ RT Master Mix (Takara Bio Inc., Shiga Prefecture, Japan) for mRNA quantification. The miScript II RT kit was used to universally tag (Qiagen, Hilden, Germany) for miRNA quantification. Quantitative PCR (qPCR) was performed using the SYBR Green PCR kit protocol and using an AB7500 RT-PCR instrument (Thermo Fisher Scientific). The relative gene expression was normalized to HPRT levels for mRNA, or U6 for miRNA using the comparative threshold cycle (2^−ΔΔCT^) method. The PCR primer sequences are presented in Supplementary Table 1.

### Dual-luciferase reporter assay

The miR-222 (miR-222-5p) target mRNA candidates were predicted using three online tools, including TargetScan [33] (http://www.targetscan.org/), miRDB [34] (http://mirdb.org/) and micro-T [35] (http://diana.imis.athena-innovation.gr/DianaTools/index.php). The predicted binding site within the NUMB mRNA 3′UTR (untranslated region) and the corresponding mutants were cloned into a luciferase-expressing vector psiCHECK2 (Promega, Madison, WI, USA). The luciferase-expressing vectors, with miR-222 mimics or scramble control (miR-NC), were co-transfected into ARPE-19 cells. Cells were harvested 48 hours after transfection. Cells were subjected to luciferase activity analysis using a dual-luciferase assay kit (Promega) and a GloMax luminometer (Promega), per manufacturer’s instructions. *Renilla* luciferase activity was measured and normalized to firefly luciferase activity, and the relative luciferase activity compared to the control group was calculated.

### Western blot assay

ARPE-19 cells were lysed in radioimmunoprecipitation assay buffer (Beyotime, Shanghai, China) supplemented with proteinase inhibitors and phosphatase inhibitors (Selleck, Houston, TX, USA). Equal amounts of protein (30 μg) were loaded and separated on 10% sodium dodecyl sulfate-polyacrylamide gel (SDS-PAGE) and transferred to polyvinylidene fluoride (PVDF) membranes (EMD Millipore, Billerica, MA, USA). The membranes were blocked with 5% nonfat milk and were immunoblotted with primary antibodies at 4°C overnight. After washing in PBS-T (PBS containing 0.1% Tween-20) three times, the membranes were incubated with horseradish peroxidase (HRP)-labeled secondary anti-rabbit or anti-mouse antibodies for 1 h at room temperature. Proteins of interest were visualized using an enhanced chemiluminescence kit (EMD Millipore), and band intensities were quantified by densitometry using ImageJ software (National Institutes of Health, Bethesda, MD, USA). More detailed information on the antibodies used for western blot assays is listed in Supplementary Table 2.

### Statistics

Data are represented as the means standard ± deviation (SD) of at least three separate experiments. Differences between the means were determined using a student’s *t*-test or a one-way analysis of variance, followed by Dunnett’s post hoc test for multiple comparisons. The differences were considered to be significant at *P* < 0.05. GraphPad Prism 5.0 software (GraphPad Software, San Diego, CA, USA) was used for statistical analyses.

### Data availability

The datasets used and/or analyzed during the current study are available from the corresponding author on reasonable request.

## RESULTS

### Palmitic acid promotes human retinal pigment epithelial cell migration

**Figure 1:**
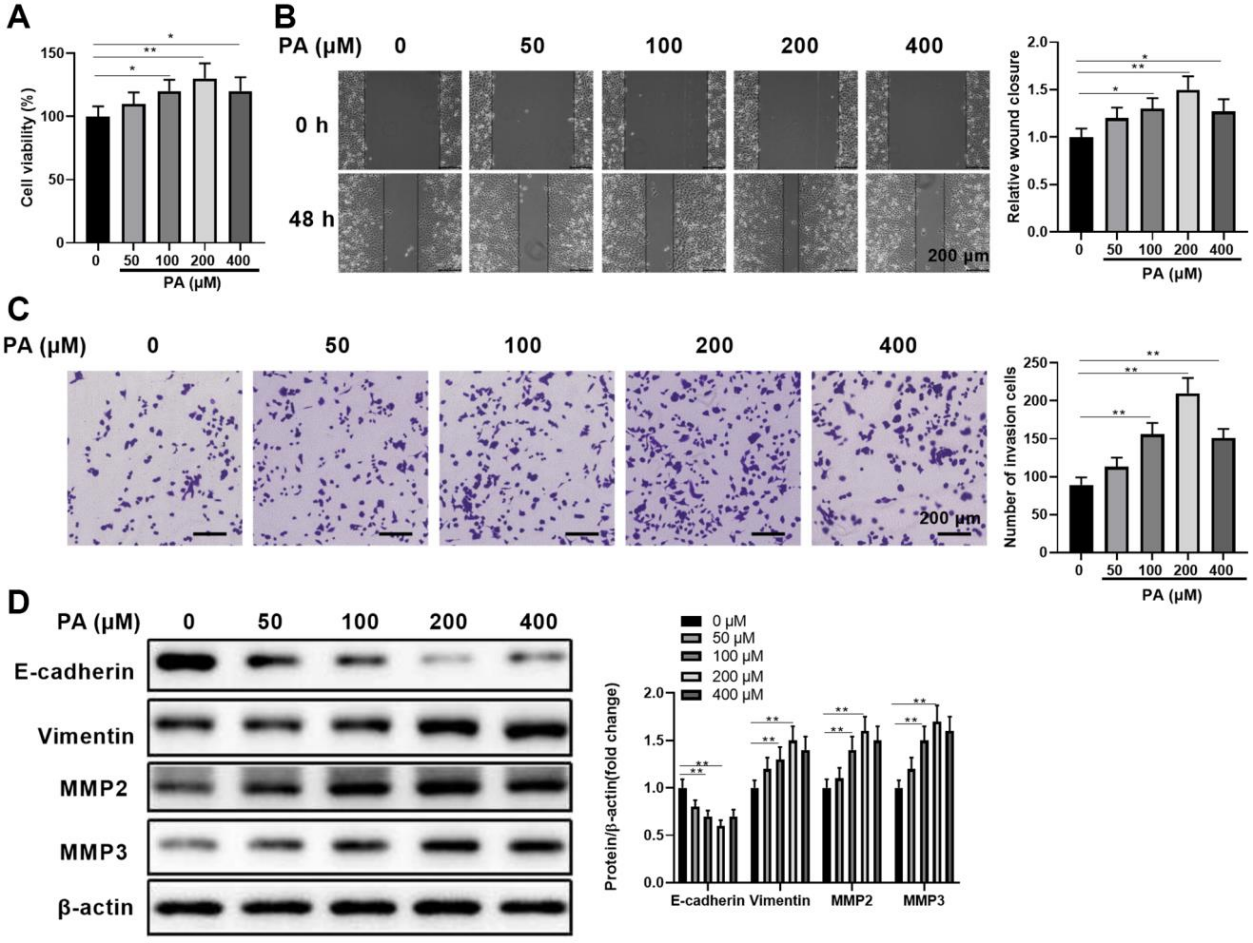
**Palmitic acid promoted human retinal pigment epithelial cell migration.** (**A**) ARPE-19 cell viability when treated with palmitic acid (PA) at the indicated concentrations for 48 h. (**B**) ARPE-19 cell wound healing ability when treated with palmitic acid at the indicated concentrations. Representative images of cells before (0 h) and after (48 h) the scratch are shown, and the relative wound closure was summarized. Scale bar, 200 μm. (**C**) ARPE-19 cell migration measured by transwell migration assays. Representative images are shown, and the invaded cell numbers per view field were summarized. Scale bar, 200 μm. (**D**) EMT-associated protein levels in the indicated ARPE-19 cells. Representative bands images are shown, and the relative protein levels were quantitated. *n* = 3 for each group; ^*^P < 0.05, ^**^P < 0.01, between the indicated groups.

To assess the effects of PA on human RPEC migration, we first evaluated the impacts of PA on ARPE-19 cell viability. As shown in Figure 1A, PA increased APRE-19 cell viability in a dose-dependent manner when administrated at a dose range of 0–200 μM. Compared with the control cells without PA treatment, APRE-19 cells treated with 400 μM PA still had increased cell viability, albeit lower than cells treated with 200 μM PA (Figure 1A). This suggests possible PA cytotoxicity at very high doses. Next, we determined the direct effects of PA at varying doses on APRE-19 cell migration by scratch healing assay (Figure 1B) and transwell migration assay (Figure 1C). Similarly, PA administrated at the doses between 0 μM and 200 μM increased wound closure (Figure 1B) and the numbers of invaded cells (Figure 1C) in a dose-dependent manner, while PA administrated at 400 μM caused a slight decrease in migration in comparison to 200 μM PA. We also examined how PA affected ARPE-19 cell EMT *in vitro* by quantitating the EMT-associated protein levels E-cadherin, vimentin, MMP2, and MMP3. As expected, palmitic acid-enhanced ARPE-19 cell migration was positively correlated with increased E-cadherin protein expressions. However, a clear inverse relationship was seen between vimentin and E-cadherin expression (Figure 1D). In addition, MMP2/MMP3 protein levels also decreased upon PA treatment with varying doses (Figure 1D), further supporting the dose-dependent PA effect on promoting EMT. Collectively, these results demonstrated that PA can significantly enhance APRE-19 cell migration, which was associated with EMT induction.

### Palmitic acid increases RPEC miR-222 expression, and miR-222 promotes RPEC migration

**Figure 2:**
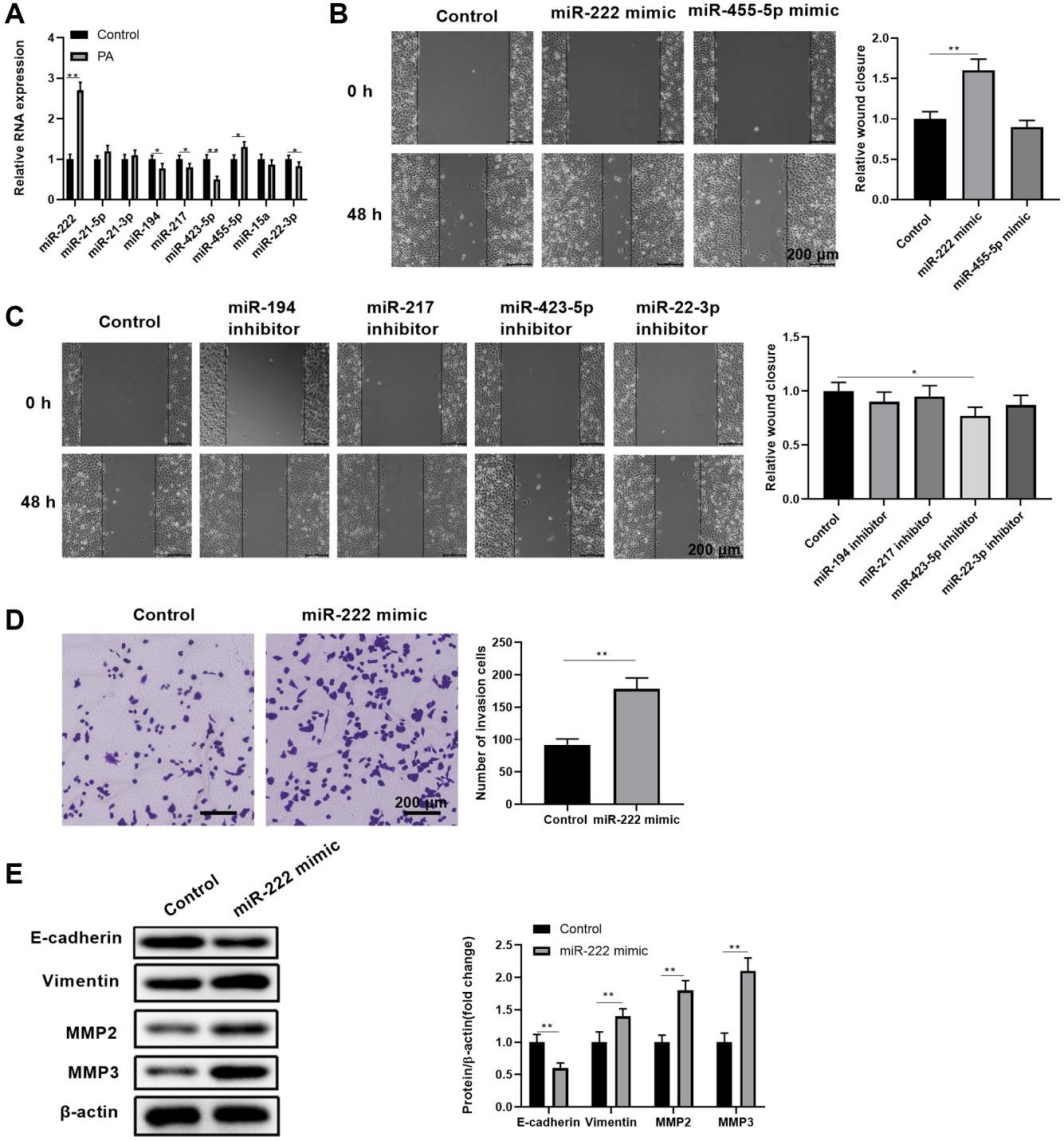
**Palmitic acid led to upregulated miR-222expression, which contributed to enhanced RPEC migration and EMT.** (**A**) The transcript levels of the indicated miRNAs in control ARPE-19 cells and ARPE-19 cells after treatment with 200 μM PA for 48 h. (**B**, **C**) The wound healing ability of ARPE-19 cells transfected with control or the indicated miRNA mimics (**B**), or transfected with control or the indicated miRNA inhibitors (**C**), was measured by scratch assays. Representative images of cells before (0 h) and after (48 h) the scratch are shown, and the relative wound closure was summarized. Scale bar, 200 μm. (**D**) The migration of ARPE-19 cells transfected with control or miR-222 mimics was determined by transwell migration assays. Representative images are shown, and the invaded cell numbers per view field were summarized. Scale bar, 200 μm. (**E**) EMT-associated protein levels in indicated ARPE-19 cells. Representative bands images are shown, and the relative protein levels were quantitated. *n* = 3 for each group; ^*^P < 0.05, ^**^P < 0.01, between the indicated groups.

Since gene regulation by miRNAs is critically involved in RPEC pathogenic conditions [23], we explored the potential miRNAs that were linked with PA in promoting RPEC migration. Among the selected 9 candidate miRNAs that were associated with palmitic acid-related fatty acids and glucose metabolisms [36–39], we found that miR-222 and miR-455-5p were significantly expressed more in ARPE-19 cells upon PA treatments, while miR-194, miR-217, miR-423-5p, and miR-22-3p were downregulated (Figure 2A). In addition, transient upregulating miR-222 levels through miRNA mimics transfection led to significantly enhanced wound closure in APRE-19 cell scratch assays (Figure 2B). This did not occur with transient upregulating miR-455-5p levels. Moreover, functionally blocking single miRNAs through miRNA inhibitor transfection, including miR-194, miR-217, miR-423-5p, and miR-22-3p, did not result in enhanced APRE-19 cell migration (Figure 2C). Therefore, we focused on miR-222 to evaluate how its ectopic expression could change ARPE-19 cell migration and EMT phenotypes. Compared with the control miRNA mimic, miR-222 mimics significantly increased the number of invaded cells in the transwell migration assay (Figure 2D). In addition, miR-222 also caused reduced E-cadherin expression and increased vimentin, MMP2, and MMP3 protein levels (Figure 2E). Taken together, these data indicated that PA increases miR-222 expression to enhance APRE-19 cell migration and EMT initiation.

### miR-222 inhibition reverses the palmitic acid’s promoting effects on RPEC migration

**Figure 3:**
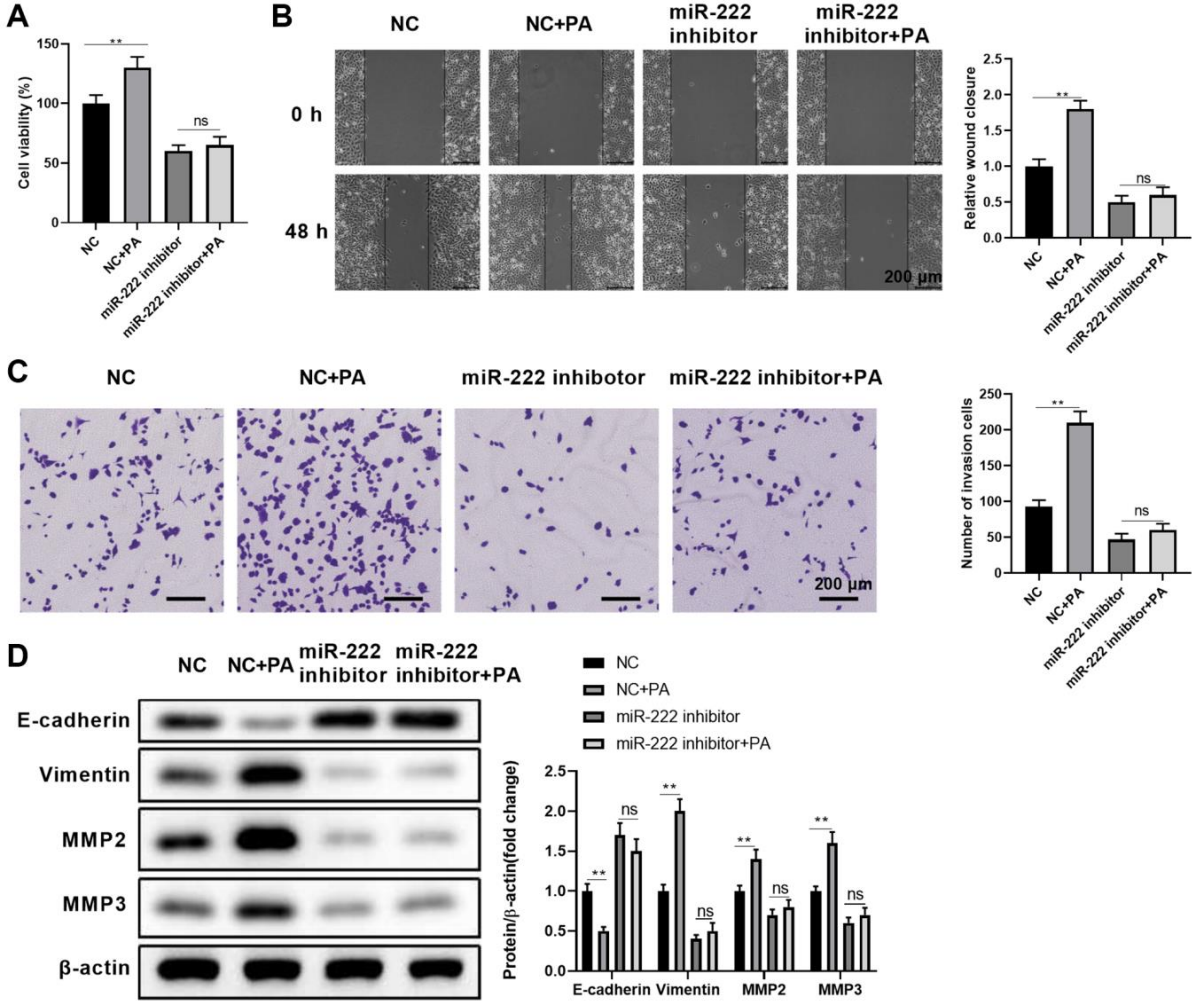
**Functional miR-222 blockade reversed the palmitic acid promoting effects on RPEC migration.** (**A**–**D**) ARPE-19 cells were transfected with control (NC) or miR-122-specific inhibitors. After 48 h, cells were left untreated, or further treated with 200 μM PA for another 48 h during the indicated assays: (**A**) Cell viability, (**B**) wound healing ability, and (**C**) migration of the indicated ARPE-19 cells. Representative images are shown on left, and the relative wound closure (**B**) and numbers of invaded cells (**C**) were summarized. Scale bar, 200 μm. (**D**) The protein levels of E-cadherin, vimentin, MMP2, and MMP3 in the indicated ARPE-19 cells. Representative bands images are shown, and the relative protein levels were quantitated. *n* = 3 for each group; ^*^P < 0.05, ^**^P < 0.01. Abbreviation: ns: not significant, between the indicated groups.

To further substantiate miR-222’s contribution to enhanced palmitic acid-mediated RPEC migration, we examined whether functionally blocking miR-222 could erase the effects of PA. First, we compared the impacts of single PA administration, single miR-222 mimic transfection, and simultaneous miR-222 mimic transfection and PA administration on ARPE-19 cell viability. As shown in Figure 3A, compared with the respective negative controls, functional miR-222 blockade alone or in combination with PA treatment significantly reduced ARPE-19 cell viability. Moreover, miR-222 blockade significantly reduced wound closure in the scratch healing assay (Figure 3B) and the numbers of invaded cells in the transwell migration assay (Figure 3C), even when PA was administrated at the same time. Furthermore, palmitic acid-mediated downregulation of E-cadherin and upregulation of vimentin, MMP2, and MMP3 were reversed by additionally blocking miR-222 (Figure 3D). Therefore, functional miR-222 blockade suppressed the PA promoting effects on RPEC migration and EMT.

### NUMB is a downstream target of miR-222 in human RPECs

**Figure 4:**
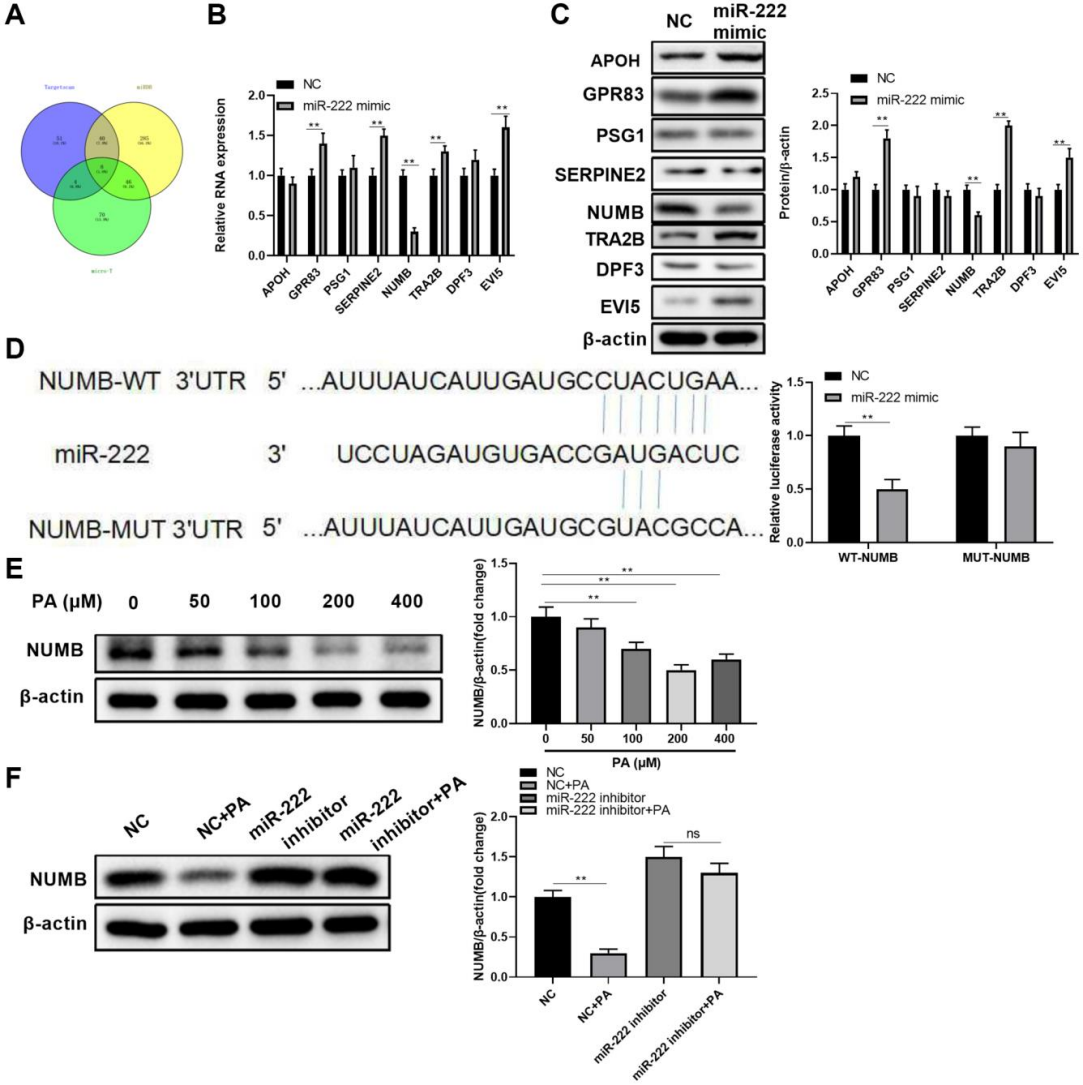
**NUMB is a direct miR-222 target in human retinal pigment epithelial cells.** (**A**) A Venn diagram shows the numbers of candidate miR-222 mRNA targets predicted by three public algorithms: TargetScan, miRDB, and micro-T. (**B**) The mRNA and (**C**) protein levels of the indicated candidates in ARPE-19 cells were determined 48 h after transfection of control (NC) or miR-222 mimics. (**D**) Diagrams show the putative miR-222 binding sites and corresponding wild type (WT) or mutant (MUT) sites of NUMB 3’UTR. The luciferase activity was detected in ARPE-19 cells at 48 h after co-transfection of WT or MUT NUMB 3’UTR together with negative control (NC) or miR-222 mimics. (**E**) NUMB protein levels in ARPE-19 cells treated with palmitic acid at the indicated concentrations. (**F**) ARPE-19 cells were transfected with negative control or miR-222 mimics, and 48 h later cells were left untreated or treated with 200 μM palmitic acid for another 48 h. NUMB protein levels in each group were determined by western blot. For all western blot data, representative bands images are shown, and the relative protein levels were summarized from three independent experiments with similar results. *n* = 3 for each group; ^*^P < 0.05, ^**^P < 0.01. Abbreviation: ns: not significant, between the indicated groups.

To further investigate the underlying miR-222 mechanisms in suppressing RPEC migration, we utilized public algorithms, including TargetScan, miRDB, and micro-T, to predict potential miR-222 target mRNAs. As shown in Figure 4A, eight genes were putative miR-222 target mRNAs, as concluded from all the three algorithms. However, among these eight common candidates, only NUMB transcript levels were significantly downregulated in ARPE-19 cells after miRNA mimic transfection (Figure 4B). Additionally, miR-222 mimic transfection in ARPE-19 cells also resulted in significant NUMB protein reduction, but not other candidate proteins (Figure 4C). Therefore, we selected NUMB as the miR-222 downstream target and further examined their relationship by luciferase reporter assays. A sequence located at the 3′UTR of NUMB was highly complementary with the miR-222 seed sequence. The luciferase activity was significantly decreased after co-transfection with wild type NUMB 3′UTR and miR-222 mimics. However, luciferase activity did not significantly change after co-transfection with mutated NUMB 3′UTR and miR-222 mimics in ARPE-19 cells (Figure 4D), implying miR-222 could no longer direct bind to the predicted 3′UTR of NUMB. In addition, PA reduced the NUMB protein levels in ARPE-19 cells in a dose-dependent manner, and peak reduction was observed when PA was administrated at 200 μM (Figure 4E). Furthermore, functionally blocking miR-222 alone or in combination with PA administration profoundly increased NUMB protein levels in ARPE-19 cells (Figure 4F). These results suggest that NUMB is a downstream target of miR-222 in human RPECs.

### NUMB knockdown abolishes palmitic acid’s promoting effects on RPEC migration

**Figure 5:**
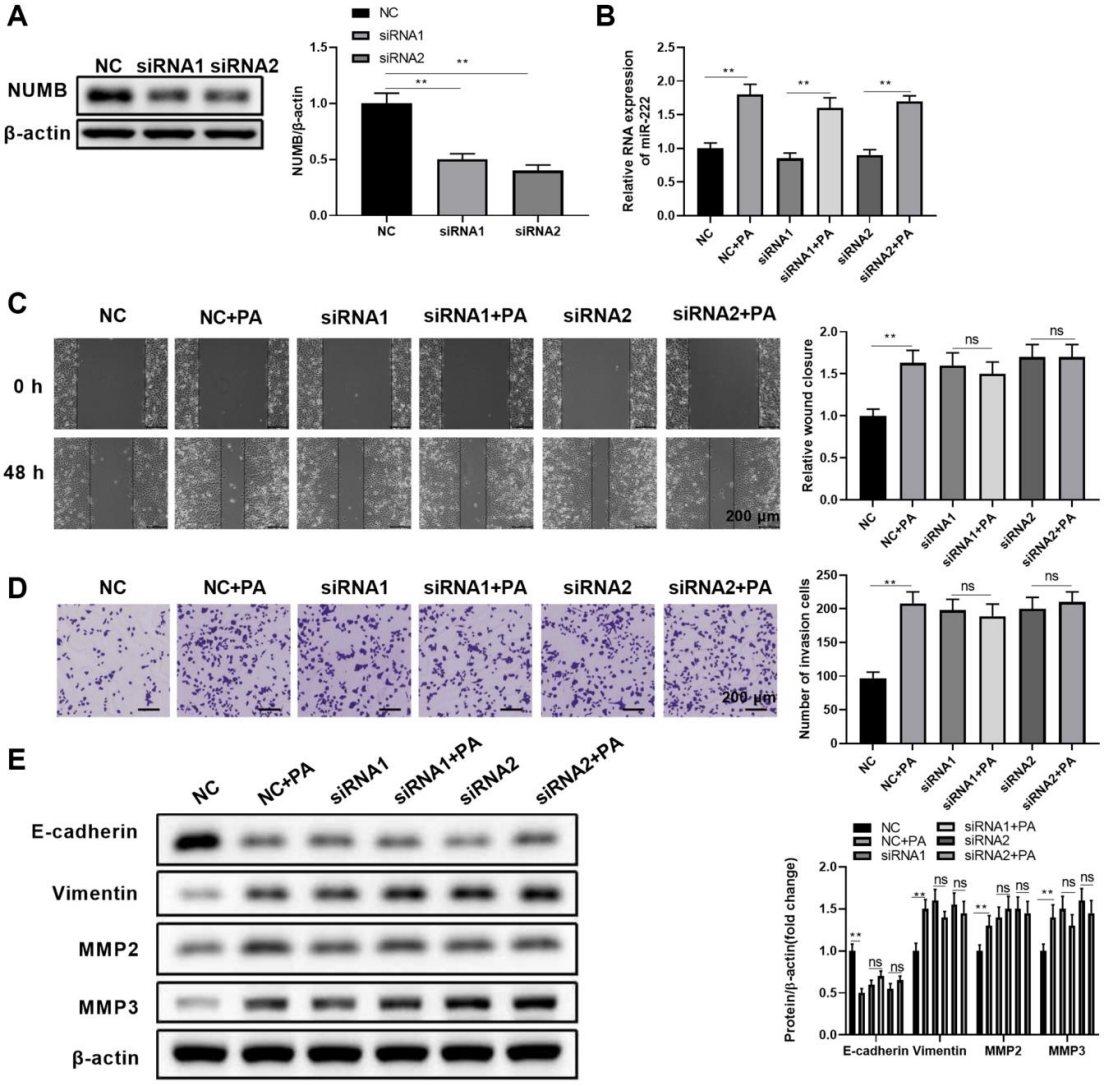
**NUMB knockdown abolished the palmitic acid promoting effect in RPEC migration.** (**A**) NUMB protein levels in ARPE-19 cells at 48 hours after transfection of negative control (NC) or NUMB-specific siRNA (siRNA1 and siRNA2) oligos. (**B**–**E**) ARPE-19 cells were separately transfected with NC, siRNA1, and siRNA2. After 48 h, cells were left untreated or treated with 200 μM palmitic acid for another 48 h in the subsequent assays. (**B**) miR-222 levels in the indicated cells after palmitic acid administration for 48 h. (**C**) Wound healing ability and (**D**) migration ability of the indicated ARPE-19 cells. (**E**) E-cadherin, vimentin, MMP2, and MMP3 protein levels in the indicated ARPE-19 cells. Representative images are shown, and the summarized data were from three independent results. *n* = 3 for each group; ^*^P < 0.05, ^**^P < 0.01. Abbreviation: ns: not significant, between the indicated groups.

The role of PA in promoting RPEC migration was dependent on the upregulated expression of miR-222, which caused a drastic reduction of NUMB protein expression. Thus, we further investigated whether NUMB knockdown alone can achieve similar RPEC migration effects as PA. We also tested if PA can affect migration in NUMB knockdown ARPE-19 cells. First, we confirmed that separate transfection of two siRNA oligonucleotides, siRNA1 and siRNA2, significantly reduced NUMB protein levels in ARPE-19 cells (Figure 5A). In cells transfected with either control siRNA oligos or NUMB-specific siRNA oligos, the miR-222 levels remained the same, while PA treatment dramatically upregulated miR-222 levels regardless of NUMB expression levels (Figure 5B). Subsequent scratch healing assays (Figure 5C) and transwell migration assays (Figure 5D) demonstrated that NUMB knockdown ARPE-19 cells had improved wound healing ability and enhanced migration compared to control cells. However, additional PA treatment with these cells did not cause further enhancement in RPEC migration. Similarly, NUMB silencing with either siRNA1 or siRNA2 significantly reduced E-cadherin and increased vimentin, MMP2, and MMP3 protein levels. Additional PA did not result in further changes in the EMT-associated protein levels (Figure 5E). Therefore, our results indicated that knockdown of the downstream effector molecule, NUMB, completely abolished the PA’s promoting effects on RPEC migration.

## DISCUSSION

**Figure 6:**
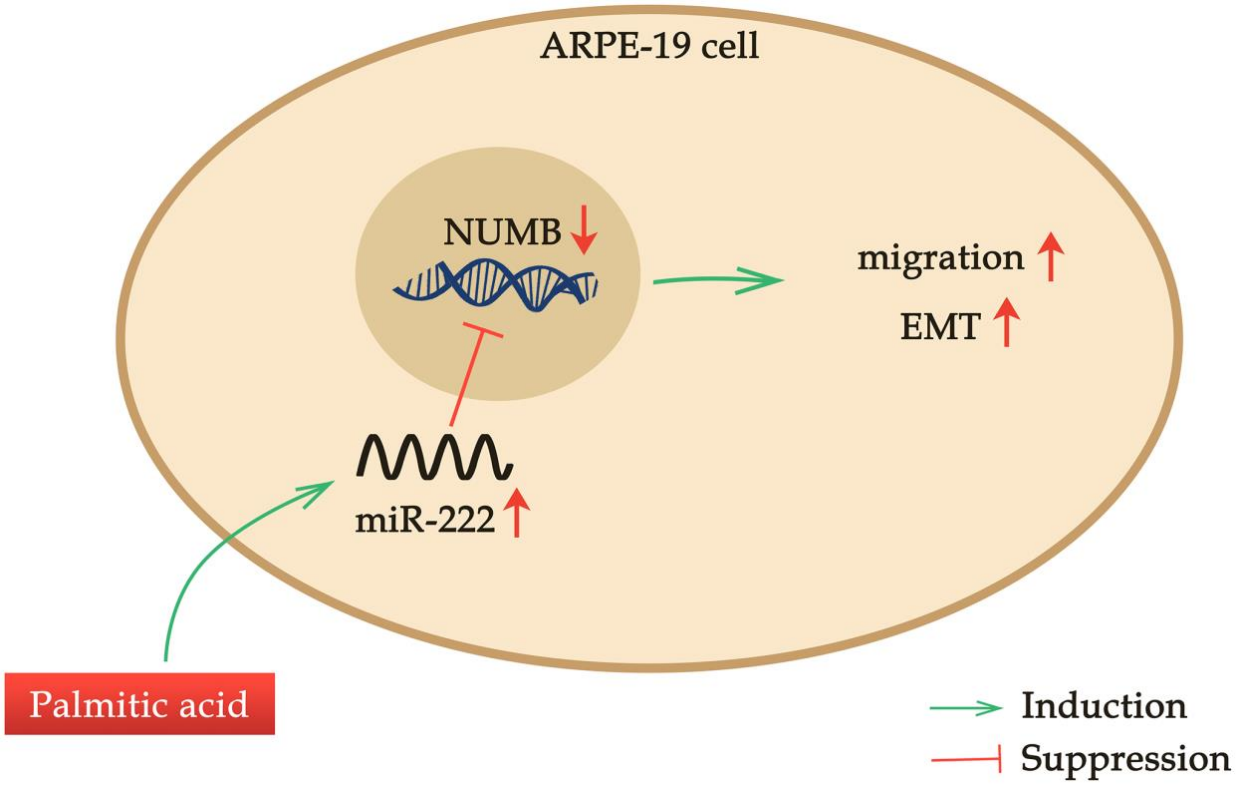
**A novel palmitic acid-miR-222-NUMB pathway in regulating human RPEC migration.** PA promotes ARPE-19 cell migration in a dose-dependent manner when administrated at a concentration no more than 200 μM. PA induces miR-222 expression and promotes the EMT of ARPE-19 cells. NUMB is one downstream target of miR-222. PA treatment suppresses NUMB expression, and NUMB knockdown alone in ARPE-19 cells also enhances RPEC migration and EMT.

RPEC migration is an important step in the pathogenesis of various ocular diseases, in which RPEC proliferation and migration are dysregulated in response to various stimulants [5, 6]. Here, we found that PA, the most abundant FFA in the human body, enhanced ARPE-19 cell migration in a dose-dependent manner when administrated at a concentration no more than 200 μM. Additionally, PA promoted RPEC EMT. Palmitic acid induced miR-222 expression, which was required for the PA’s promoting effects on ARPE-19 cell migration. NUMB was identified as the downstream target of miR-222, and NUMB expression was suppressed in a dose-dependent manner by PA treatments. Moreover, NUMB knockdown alone in ARPE-19 cells recapitulated the treatment of PA in mediating RPEC migration and EMT, as well as erased the promoting effects of additional PA treatment on RPEC migration and EMT. Thus, our study revealed a novel palmitic acid-miR-222-NUMB pathway in regulating human RPEC migration (Figure 6).

Uncontrolled RPEC proliferation and migration can form pathological membranes on both neural retina surfaces, eventually resulting in visual impairment [7]. For instance, RPEC migration to the ectopic sites and subsequent membrane formation plays an important role in diabetic retinopathy pathogenesis [6]. Therefore, RPEC migration suppression may represent a rational strategy for developing novel therapeutics for patients with diabetic retinopathy. However, this strategy may not be the case for aged patients with AMD. AMD typically lacks RPEC migration and the followed replacement of dying REPCs, as well as excessive intraretinal RPEC migration into the vitreous and on the retinal surface, and these pathologies might occur simultaneously to control the disease progression [8–10]. Therefore, identifying factors that can modulate RPEC migration is critical for many ocular disease therapies. We found that PA promotes RPEC migration, provides experimental evidence for lipotoxicity-induced RPEC dysfunction, and implies a potential therapeutic strategy by targeting the miR-222-NUMB axis for protecting RPECs against lipotoxicity and lipid-related metabolic diseases.

Previous studies have revealed that different nutrient or metabolic status, oxidative stress, inflammatory environments, as well as many signaling pathways and genes, contributed significantly to regulating RPEC migration. For example, Protein Kinase C (PKC)-mediated signaling pathway plays a crucial role in human RPEC migration and is a potential therapeutic target against RPEC migration and PVR disease [40]. Consistently, PA and PKC-mediated signaling pathways were reported to work in concert to induce autophagy [41], indicating that these two factors might also co-operate in promoting RPEC migration. However, the interactions of PA with PKC signaling or other RPEC migration-related factors in human RPECs remain to be explored. In addition, PKC-mediated signaling pathway was reported to regulate gene expression of microRNAs in human T lymphocytes [42], human hepatoma HepG2 cells [43], and human keratinocytes [44, 45]. Thus, the potential interactions between PA and PKC signaling might help explain the induced expression of miR-222 by PA in ARPE-19 cells. Furthermore, reports showed that elevated FFA uptake promotes EMT in multiple malignant cell types [46, 47]. Palmitic acid also dose-dependently enhanced EMT of ARPE-19 cells, suggesting a possible conserved link between lipid metabolism and EMT in multiple cell types.

We showed that both miR-222 and miR-455-5p were upregulated in ARPE-19 cells upon PA treatments. However, only miR-222 was found involved in regulating RPEC migration. This might be due to that miR-455-5p modulated functions other than migration or EMT. Alternatively, the ability of miR-455-5p in migration regulation was too weak, and it could not be reflected under our current experimental conditions. Murine miR-222 was previously identified as a RPE signature miRNA by profiling micro-dissected RPE from wild type and *Dicer1* conditional knockout mice using Affymetrix GeneChip miRNA arrays [48]. Here, through miRNA mimic-mediated gain-of-function and miRNA inhibitor-mediated loss-of-function studies, we demonstrated miR-222 promoted ARPE-19 cell migration and EMT. First discovered in human umbilical vein endothelial cells, miR-222 expression is frequently increased in epithelial tumors [49]. miR-222’s proliferative role has been confirmed in multiple malignancies, including breast cancer, hepatocellular carcinoma, pancreatic cancer, glioblastomas, and lung cancer [49, 50]. miR-222 overexpression in hepatocellular carcinoma [26], osteosarcoma [27], and ovarian carcinoma [28] promote cell migration and invasion. Moreover, miR-222 contributes to the aggressive clinical behavior in basal-like breast cancers through targeting Trichorhinophalangeal 1 to promote EMT [29]. These results are consistent with our observations with miR-222’s role in RPEC proliferation, migration, and EMT. Our data suggest that miR-222 plays oncogenic roles in tumor initiation and development, as well as the pathogenic roles in non-cancerous cells under many physiological and pathological conditions, including palmitic acid-induced lipotoxicity.

We identified NUMB as a novel direct miR-222 target in human RPECs. An inverse correlation between NUMB protein expression and the extent of cell migration was observed, which indicates NUMB’s suppressive role in RPEC cellular processes, like migration and EMT. The new NUMB functions in RPECs uncovered here are consistent with NUMB’s role as a tumor suppressor in a variety of malignancies. One example is observed in ovarian cancer, in which NUMB inhibits cell proliferation, invasion, and EMT [51]. These findings also suggest NUMB is functionally conserved between malignant cells and non-malignant cells in terms of controlling cellular behaviors. Interestingly, NUMB can regulate additional signaling pathways in malignancies, like suppressing PAK1/β-catenin signaling pathway in ovarian cancer [51] or inhibiting notch signaling and stabilizing p53 in breast cancer [52]. Whether these pathways are also important for human RPECs remains to be explored. Taken together, our study broadens our understanding of miR-222 and NUMB roles in human RPECs under normal and palmitic acid-mediated pathological conditions. This study suggests that the miR-222-NUMB axis might be a potential novel biomarker or therapeutic target for certain ocular diseases, like PVR. Prediction of miR-222 target mRNAs using multiple public algorithms resulted in many candidates, including APOH, GPR83, PSG1, SERPINE2, NUMB, TRA2B, DPF3, and EV15. However, only NUMB was validated to be downregulated in both mRNA and protein expression when miR-222 was overexpressed in ARPE-19 cells. Interestingly, miR-222 expression-induced upregulated expression of APOH, GPR83, TRA2B and EV15 was observed. It has been reported that some miRNAs could upregulate gene expression in specific cell types and conditions with distinct transcripts and proteins [53]. This suggests that micro-ribonucleoprotein (miRNP) might be involved to act in trans in promoting the target mRNAs’ expression. The mRNA expression could be activated by the direct action of miRNPs and/or could be indirectly relieved from miRNA-mediated repression by abrogating the action of repressive miRNPs [53, 54]. Therefore, whether other mRNA target candidates can be regulated by PA treatments and their roles in modulating migration and EMT remain to be investigated in our future studies.

In summary, we elaborated on a novel palmitic acid-miR-222-NUMB pathway that regulates human RPEC migration *in vitro*. Although more work with *in vivo* animal models and clinical samples can further strengthen our findings, this study provides new insight into understanding lipid metabolism-related pathogenesis in human ocular diseases.

## Supplementary Materials

Supplementary Tables
