# Supplementary material for: Palmitic acid promotes human retinal pigment epithelial cells migration by upregulating miR-222 expression and inhibiting NUMB
Source: Aging (Albany NY). 2023 Apr 13;15(18):9341–57. doi: 10.18632/aging.204647 (PMC10564421; doi:10.18632/aging.204647)
Supplement: Supplementary Tables [file aging-15-204647-s001.pdf]

SUPPLEMENTARY TABLES

Supplementary Table 1. The sequences of miRNA mimic, inhibitor, siRNA oligos and PCR primers used in this study.

Name	Sequences
miR-222 mimic	5′-AGCUACAUCUGGCUACUGGGU-3′
miR-455-5p mimic	5′-TAUGTGCCTTTGGACTACATCG-3′
NC mimic	5′-UUCUCCGAACGUGUCACGUTT-3′<br>5′-ACGUGACACGUUCGGAGAATT-3′
miR-194 inhibitor	5′-UGUAACAGCAACUCCAUGUGGA-3′
miR-217 inhibitor	5′-UACUGCAUCAGGAACUGAUUGGA-3′
miR-423-5p inhibitor	5′-UGAGGGGCAGAGAGCGAGACUUU-3′
miR-22-3p inhibitor	5′-AAGCUGCCAGUUGAAGAACUGU-3′
miR-222 inhibitor	5′-ACCCAGUAGCCAGAUGUAGCU-3′
NC inhibitor	5′-UCACAACCUCCUAGAAAGAGUAGA-3′
NUMB-siRNA-1	5′-GGACCTCATAGTTGACCAG-3′
NUMB-siRNA-2	5′-GCCUUGCAAUUAGGCUAAATT-3′
NC-siRNA	5′-CCTAAGGTTAAGTCGCCCTCGCTC-3′
APOH	F: 5′-ATCTGCCCTCTCACAGGACT-3′<br>R: 5′-GTCGTATAGCGTACGGCTCC-3′
GPR83	F: 5′-TTGCCCAGTACTGCTCACTG-3′<br>R: 5′-AGAACGTAGCCATGGTCCAG-3′
PSG1	F: 5′-GACTCCAGACGCAAGCTACC-3′<br>R: 5′-GCACTCACTGGGTTCCGTAT-3′
SERPINE2	F: 5′-CTTTGAGGATCCAGCCTCTG-3′<br>R: 5′-TGCGTTTCTTTGTGTTCTCG-3′
NUMB	F: 5′-CCAGAAGAUGUCACCCUUUTT-3′<br>R: 5′-AAAGGGUGACAUCUUCUGGTT-3′
TRA2B	F: 5′-CGGCGAGCGGGAATCCCG-3′<br>R: 5′-GACATGGGAGAATGGCTGTGGC-3′
DPF3	F: 5′-TCGGGGACCAGTTCTACAAG-3′<br>R: 5′-GATCTTCAGGTGGGTGCAAT-3′
EVI5	F: 5′-TGGGCTGGTCTCGAACTCCTAAC-3′<br>R: 5′-GCAGTGGCTCACACCTGTAATCC-3′
HPRT	F: 5′-CATTTGTCGATGCTCAATCC-3′<br>R: 5′-TCAGTTGAAGTCATTGATGATGG-3′
miR-222	F: 5′-ACA CTC CAG CTG GGA GCT ACA TCT GGC TAC TG-3′<br>R: 5′-CTC AAC TGG TGT CGT GGA-3′
miR-21-5p	F: 5′-TAGCTTATCAGACTGATGTTGA-3′<br>R: 5′-AGTGCGTGTCGTGG-3′
miR-194	F: 5′-CACGCATGTAACAGCAAC-3′<br>R: 5′-CCAGTGCAGGGTCCGAGGTA-3′
miR-217	F: 5′-TAC TCA ACT CAC TAC TGC ATC AGG A-3′<br>R: 5′-TAT GGT TGT TCT GCT CTC TGT GTC-3′
miR-423-5p	F: 5′-ACACTCCAGCTGGGTGAGGGGCAGAGAGCGA-3′<br>R: 5′-CTCAACTGGTGTCGTGGAGTCGGCAATTCAGTTGAGAAAGTC-3′
miR-21-3p	F: 5′-GGGGCAACACCAGTCGATG-3′<br>R: 5′CAGTGCGTGTCGTGGAGT-3′
miR-455-5p	F: 5′-CGAGCTTCCTTCTGCAGGT-3′

	R: 5′-CACCACTGCCATCCCACA-3′
miR-15a	F: 5′-GGGGTAGCTTATCAGACTG-3′<br>R: 5′-AGTGCGTGTCGTGGAGTC-3′
miR-22-3p	F: 5′-AAGCTGCCAGTTGAAGAACTGTA-3′<br>R: 5′-TTACCTAGCGTATCGTTGAC-3′
U6	F: 5′-AGA GAA GAT TAG CAT GGC CCC TG-3′<br>R: 5′-ATC CAG TGC AGG GTC CGA GG-3′

Abbreviations: F: Forward; R: Reverse.

Supplementary Table 2. The information of antibodies used for western blot assays in this study.

Name	Company	Cat No.
E-cadherin	Sino Biological, Beijing, China	10204-H08H1
Vimentin	Sino Biological, Beijing, China	10028-H08B
MMP2	Sino Biological, Beijing, China	10082-HNAH
MMP3	Sino Biological, Beijing, China	10467-HNAE
NUMB	Sino Biological, Beijing, China	100576-T36
APOH	Santacruze, California, USA	sc-134264
GPR83	FineTest, Wuhan, China	FNab03611
PSG1	Abcam, Cambridge, UK	Ab233130
SERPINE2	FineTest, Wuhan, China	FNab07752
TRA2B	bioss, Beijing, China	bs-17180R-1
DPF3	Sino Biological, Beijing, China	102918-T08
EVI5	AtaGenix, Wuhan, China	ATA28904
Beta-actin	Sino Biological, Beijing, China	12269-H08H
Anti-rabbit 2nd	Invitrogen, California, USA	DXT-081094
Anti-mouse 2nd	Invitrogen, California, USA	081342
